# Amphotericin B Formulations and Other Drugs for Visceral Leishmaniasis

**DOI:** 10.4269/ajtmh.14-0743

**Published:** 2015-03-04

**Authors:** Jonathan Berman

**Affiliations:** Fast Track Drugs and Biologics, North Potomac, Maryland

Visceral leishmaniasis (VL), which is endemic in the Indian subcontinent (42,623 reported cases/year of which 34,918 were in India), the Mediterranian region (875 cases/year), East Africa (8,569 cases/year), and Brazil (3,481 cases/year)^1^ has undergone a revolution in chemotherapy in the last 15 years. Treatment had been with the classical agents pentavalent antimony and amphotericin B deoxycholate, but with > 90% of Indian disease occurring in antimony-resistant regions,^2^ the sole effective drug in this key region of the world was amphotericin B deoxycholate. Amphotericin B deoxycholate is extraordinarily effective for Indian VL. In phase 3 studies using 1 mg/kg every other day for 15 intravenous injections, 259 of 260 (99.6%) of per-protocol patients were cured (Table 1 regimens 1 plus 2).^3^,^4^ Nevertheless, amphotericin B deoxycholate toxicity (infusion-related fever and chills, an infusion-related cardiovascular-respiratory syndrome, which can be mortal, renal and hematologic reactions), and the considerable inconvenience of 15 intravenous injections led to strenuous efforts in the 2000s, many by Dr. Sundar and associates in the Indian subcontinent, to find a replacement that is as effective and inexpensive (< $500 per course)^5^ but less toxic and less difficult to administer.

One approach is to use a different formulation of amphotericin B that might diminish toxicity, thereby permitting larger individual doses and a shorter total treatment period. Most reports concern liposomal amphotericin B (Ambisome), but there are a few reports on amphotericin B lipid complex (ABLC/Abelcet), amphotericin B colloidal dispersion (ABCD/Amphocil), amphotericin B lipid emulsion (ABLE), and in this issue of the *Journal*, an Indian-formulated liposomal amphotericin B (Fungisome).

Ambisome: Although adverse reactions to Ambisome are qualitatively similar to those of amphotericin B deoxycholate, their frequency and severity are diminished.^6^ Improved tolerance led to higher daily doses, a trial of 7.5 mg/kg total dose over 5 days, and then a trial of 7.5 mg/kg in merely one injection, but both regimens were only 90–93% effective (Table 1 regimens 3 and 4).^7^,^8^ To bring efficacy up to amphotericin B deoxycholate levels, the total dose of Ambisome was increased to 10 mg/kg. Both 10 mg/kg over 5 days and finally 10 mg/kg administered once showed a high efficacy rate of 96% (Table 1 regimens 5 and 6).^5^,^9^

ABLC/Abelcet: In a head-to-head comparison, Abelcet was inferior to Ambisome in efficacy (Table 1 regimen 7 versus regimen 5) and in tolerance (fever/chills were experienced by 76% of ABLC and 29% of Ambisome patients).^5^

ABCD/Amphocil: 97% efficacy was shown in a large trial using < 1 week of therapy (Table 1 regimen 8).^10^

ABLE: 15 mg/kg in one injection was only 85% effective (Table 1 regimen 9).^11^

Fungisome: In this issue of the *Journal*, Sundar and others report the efficacy of one injection of 10 mg/kg or of 15 mg/kg in an early phase 2 study of 15 patients per cohort.^12^ One patient in each cohort relapsed therefore the cure rate was 14 of 15 (93%) for each regimen (Table 1 regimen 10). There was a 90% incidence of infusion-related fever and chills, and an ∼25% incidence of diarrhea and vomiting. The 5 SAEs (2 nephrotoxicity, 2 thrombocytopenia, 1 pulmonary edema), even though reversible, may be considered frequent for a small 30-patient database.

In sum, systematic evaluation of Ambisome has led to a very high dose of 10 mg/kg administered in a very short period of time of 1 day, which in a large study showed 96% efficacy. This regimen is overall superior to the standard regimen of amphotericin B deoxycholate (1 mg/kg every other day for 15 infusions) on the basis of efficacy (almost equal), tolerance (superior), feasibility (far superior), and cost (∼$200 for Ambisome at the developing-world favorable price). Ambisome 10 mg/kg once is now the treatment of choice for Indian subcontinent VL.^12^,^13^ ABCD/Amphocil showed excellent efficacy in a relatively short course; whether single dose Amphocil is competitive with single dose Ambisome is not known. Other amphotericin B formulations were either inferior in efficacy (ABLC/Abelcet, ABLE) or have not yet been evaluated in large trials (Fungisome).

Other approaches toward replacing amphotericin B deoxycholate for Indian subcontinent VL are a parenteral agent that can be administered intramuscularly (paromomycin) and an oral agent (miltefosine).

In a large phase 3 study, 95% of Indian per-protocol patients were cured with a regimen of paromomycin 11 mg/kg/day for 21 days intramuscularly (Table 1 regimen 11).^4^ An attempt to shorten the inconvenient 21-day intramuscular treatment course to 14 days revealed low efficacy (84%) for the 2-week course (Table 1 regimen 12).^14^

In a large phase 3 trial, 97% of Indian per-protocol patients were cured with a regimen of miltefosine 2.5 mg/kg/day for 28 days (Table 1 regimen 13)^3^ after which miltefosine was made the VL treatment of choice in India, but after 10 years of use, the efficacy rate has fallen to 90% (Table 1 regimen 14).^15^ Gastrointestinal side effects are frequent, and miltefosine is contraindicated in pregnancy.

Combining short courses of two drugs will decrease the length of parenteral therapy and should protect against resistance including that to miltefosine and the aminoglycoside paromomycin. When combinations of short courses of 2-drug combinations of Ambisome, miltefosine, and paromomycin were evaluated, each combination was 99% effective (Table 1 regimens 15–17) in substantial numbers of patients.^16^ Choosing between these three combinations is difficult; the main reason *not* to use a combination involving Ambisome, miltefosine, or paromomycin is the need to maintain a cold-chain, female contraception, and 10 days of injections, respectively.

A fundamental issue with anti-VL chemotherapy is that although the incidence of VL in India is such that high-quality studies can be performed, efficacy against Indian VL does not convey to disease from other endemic regions. Ambisome and paromomycin regimens that were 90% and 95% effective against *Leishmania donovani* in India (Table 1 regimens 4 and 11) were 40% and 66% effective against *L. donovani* in East Africa (Table 1 regimens 18 and 20). Even 21 mg/kg Ambisome was only 85% effective in East Africa (Table 1 regimen 19),^17^ although such a dose seems effective against Mediterranian disease caused by *Leishmania infantum* (Table 1 regimen 21).^18^ On the other hand, the classic agent pentavalent antimony is still effective in East Africa (Table 1 regimen 22)^19^ and in the Mediterranian (Table 1 regimen 23).^20^ These discrepancies are important not just for non-Indian endemic regions, but for VL seen in the developed world. Visceral leishmaniasis is uncommon in the United States; however, the disease that this reviewer has seen in the US was contacted in the Mediterranian, East Africa, and Brazil. Other issues for the United States are FDA approval (Ambisome, Abelcet, and miltefosine are approved products in the United States) and pricing that will not be the same as in India.

Remarkable progress has been made in VL chemotherapy with the advent of an oral agent and then short-course Ambisome in the Indian subcontinent. The future of anti-VL chemotherapy is likely to involve further fine-tuning of lipid formulations of amphotericin B such as Fungisome to possibly compete with Ambisome in the Indian subcontinent; use of single agent approved products in the developed nations; and combination therapy worldwide, to include clinically resistant disease in patients with underlying immunodeficiencies.

## Figures and Tables

**Table 1:** Efficacy of drug regimens for visceral leishmaniasis

Regimen no.	Drug and route	Regimen*	Per-protocol efficacy 6 M after RX†	Reference
Drugs for visceral leishmaniasis in otherwise immunocompetant patients in the Indian subcontinent				
1	Amphotericin B deoxycholate IV	15 mg/kg over 30 days	96/96 (100%)	3
2	Amphotericin B deoxycholate IV	15 mg/kg over 30 days	163/164 (99%)	4
3	Liposomal amphotericin B /Ambisome IV	7.5 mg/kg over 5 days	26/28 (93%)	7
4	Liposomal amphotericin B /Ambisome IV	7.5 mg/kg once	183/203 (90%)	8
5	Liposomal amphotericin B /Ambisome IV	10 mg/kg over 5 days	49/51 (96%)	5
6	Liposomal amphotericin B /Ambisome IV	10 mg/kg once	291/304 (96%)	9
7	ABLC/Abelcet IV	10 mg/kg over 5 days	47/51 (92%)	5
8	ABCD/Amphocil IV	7.5 mg/kg over 6 days	131/135 (97%)	10
9	ABLE	15 mg/kg once	317/373 (85%)	11
10	India-formulated Liposomal Amphotericin B/Fungisome IV	10 mg/kg or 15 mg/kg once	14/15 (93%)	12
11	Paromomycin IM	11 mg/kg/d × 21 days	474/501 (95%)	4
12	Paromomycin IM	11 mg/kg/d × 14 days	183/217 (84%)	14
13	Miltefosine oral	2.5 mg/kg/day × 28 days	282/291 (97%)	3
14	Miltefosine oral	2.5 mg/kg/day × 28 days	512/567 (90%)	15
15	Ambisome IV + Miltefosine oral	5mg/kg once + 2.5 mg/kg/d for 7 days	155/157 (99%)	16
16	Ambisome IV + Paromomycin IM	5mg/kg once + 11 mg/kg/d for 10 days	153/155 (99%)	16
17	Miltefosine oral + Paromomycin IM	2.5 mg/kg/day for 10 days + 11 mg/kg/d for 10 days	156/158 (99%)	16
Drugs for visceral leishmaniasis in otherwise immunocompetant patients in other endemic regions				
18	Ambisome IV in East Africa	7.5 mg/kg once	8/20 (40%)	17
19	Ambisome IV in East Africa	21 mg/kg over 21 days	46/54 (85%)	17
20	Paromomycin in East Africa	11 mg/kg/d × 21 days	80/121 (66%)‡	19
21	Ambisome IV in Mediterranian	18 mg/kg over 10 days	41/42 (98%)	18
22	Pentavalent Antimony in East Africa	20 mg/kg/day × 30 days	104/112 (93%)‡	19
23	Pentavalent Antimony in Mediterranian	20 mg/kg/day × 30 days	47/52 (90%)	20

* mg drug = mg active ingredient (amphotericin B, paromomycin base, miltefosine, antimony) in the formulation.

† Efficacy = no. cured/no. evaluable (%).

‡ HIV-positive patients omitted from calculation.
